# Atom bond connectivity index for graph with self-loops and its application to structure property relationships in anticancer drugs

**DOI:** 10.1038/s41598-025-09789-z

**Published:** 2025-07-05

**Authors:** B. Sharath, H. J. Gowtham

**Affiliations:** Department of Mathematics, Manipal Institute of Technology, Manipal Academy of Higher Education, Manipal, 576104 India

**Keywords:** Atom bond connectivity index, Anticancer drugs, Graph with self-loops, Drug discovery, Chemistry, Mathematics and computing

## Abstract

Let $G_S$ be a graph derived from a simple graph *G* by adding a self-loop to each vertex in a subset $S\subseteq V(G)$. In this paper, we define the atom bond connectivity index of the graph $G_S$ as $ABC(G_S)$ and the atom bond connectivity energy of $G_S$ as $E_{ABC}(G_S)$. We obtained upper bounds for the *ABC* spectral radius of the graph $G_S$ as well as bounds for $E_{ABC}(G_S)$ and $ABC(G_S)$ in terms of *m*, *n*, $\Delta$ and $\delta$. Additionally, we computed the *ABC* energy for complete graph, cocktail party graph and crown graph with self-loops. We also derived the characteristic polynomial of double star graph with self-loops. Furthermore, we explored the correlation between $ABC(G_S)$ and various physico-chemical properties, such as boiling point (BP) and molar refraction (MR). Furthermore, we established correlations between $ABC(G_S)$ and specific indices, specifically the Sombor index of a graph $G_S$ $(SO(G_S))$, first Zagreb index of a graph $G_S$ $(M_1(G_S))$, and Randic index of a graph $G_S$ $(R(G_S))$.

## Introduction

Topological indices are numerical values derived from the connectivity patterns of graphs, serving as tools to extract and condense the information embedded within these patterns. In 1947, H. Wiener^1^ defined the topological indices, which allowed him to compare the boiling points of various alkane isomers. Let *G* be a simple graph and Let $G_{S}$ be a graph with self-loops obtained from a simple graph *G* by attaching self-loops to each of the vertices belonging to $S \subseteq V (G)$ with $|S| = \sigma$^2^. The maximum and minimum degrees of the graph *G* are denoted by $\Delta$ and $\delta$, respectively. The degree $d_{i}(G_{S})$ represent the number of edges incident on the $i^{ th}$ vertex of $G_{S}$ for $1 \le i \le n$.

In literature, numerous topological indices have been defined and used as molecular descriptors. For more on topological indices see^2–7^. Any degree-based topological indices of the form $TI(G)=\sum \limits _{v_i\sim v_j}f(d_i, d_j)$, where *f* is suitably chosen function and $d_i$ and $d_j$ are vertex degrees of graph G.

In 1998, a new index was proposed by E. Estrada et al.^8^, which is later referred as the atom bond connectivity index (or *ABC* index), which was developed to model the thermodynamic characteristics of organic chemical compounds. The *ABC* index of graph *G*, denoted by *ABC*(*G*), is defined as the sum of weights $\sqrt{\frac{d_i+d_j-2}{d_id_j}}$ over all edges $v_iv_j$ of G. i. e.,

$$\begin{aligned} ABC(G)=\sum \limits _{v_iv_j\in {E(G)}}\sqrt{\frac{d_i+d_j-2}{d_id_j}}. \end{aligned}$$

Initially, this paper gained minimal attention from researchers. In 2008, E. Estrada introduced a study^9^ that utilized the ABC index to analyze the stability of branched alkanes. This study sparked considerable interest among mathematicians, resulting in numerous investigations into the mathematical properties of the ABC index^10–12^. B. Furtula et al.^13^ considered a generalization of the ABC index in order to explore its better correlation capabilities regarding the heat of formation of alkanes, namely

$$\begin{aligned} ABC_\alpha (G)=\sum \limits _{v_iv_j\in {E(G)}}\left( \frac{d_i+d_j-2}{d_id_j}\right) ^\alpha . \end{aligned}$$

E. Estrada introduced a matrix known as the *ABC* matrix^14^, which is closely related to atom bond connectivity index and is commonly referred to as the *ABC* index. This matrix represents the probability of visiting a nearest neighbor edge from either side of a given edge in a graph. In the context of molecular graphs, this can be associated with the bonding capacity of the bond being analyzed. The *ABC* matrix is expressed as follows:

$$A_{ABC}(G)_{ij}= {\left\{ \begin{array}{ll} \sqrt{\frac{d_i+d_j-2}{d_id_j}}, & \hbox { if}\ v_i \sim v_j\\ {0}, & \hbox { if}\ v_i \not \sim v_j . \end{array}\right. }$$

The *ABC* energy of a graph *G* is defined as, $E_{ABC}(G)=\sum\limits_{i=1}^{n}|\lambda _i|$, where $\lambda _1, \lambda _2, \ldots , \lambda _n$ are the eigenvalues of $A_{ABC}(G)$. The eigenvalues of $A_{ABC}(G)$ satisfy the following relation^15^:

$$\begin{aligned} \sum \limits _{i=1}^{n}\lambda _i=0 \qquad \text{ and } \qquad \sum \limits _{i=1}^{n}\lambda _i^2=2\left( n-2R_{-1}(G)\right) \end{aligned}$$

where $R_{-1}(G)$ is second modified Zagreb index of a graph *G*. i. e., $R_{-1}(G)=\sum \limits _{v_iv_j\in E(G)}\frac{1}{d_id_j}$.

In the domain of spectral graph theory, energy of graph with self-loops was introduced recently by I. Gutman^2^. Adding self-loops distinguish hetero-atoms from carbon atoms in hetero conjugated molecules^16–19^, the spectral aspect of a simple graph extended to a graph with self-loops opens up a new area of study for structural features and related chemical properties of molecules. Motivated by this, we now extended the *ABC* index from a simple graph *G* to a graph $G_S$. In analogy to *ABC* index of a graph *G*, we define *ABC* index of a graph $G_S$ as,

$$\begin{aligned} ABC(G_S)=\sum \limits _{v_iv_j\in E(G)}\sqrt{\frac{d_i(G_S)+d_j(G_S)-2}{d_i(G_S)d_j(G_S)}}+\sum \limits _{v_i\in S}\sqrt{\frac{2d_i(G_S)-2}{d^2_i(G_S)}}. \end{aligned}$$

Also defined the *ABC* matrix of a graph $G_S$ as,

$$A_{ABC}(G_S)_{ij}= {\left\{ \begin{array}{ll} \sqrt{\frac{d_i(G_S)+d_j(G_S)-2}{d_i(G_S)d_j(G_S)}}, & \hbox { if}\ v_i \sim v_j\\ {0}, & \hbox { if}\ v_i \not \sim v_j \\ \sqrt{\frac{2d_{i}(G_{S})-2}{d^2_i(G_{S})}}, & \hbox {if} \ i=j \ \hbox {and} \ v_i \in S \\ {0}, & \hbox {if} \ i=j \ \hbox  {and} \ v_i \notin S . \end{array}\right. }$$

The *ABC* energy of a graph $G_S$ is defined as,

$$\begin{aligned} E_{ABC}(G_S)=\sum \limits _{i=1}^{n}\left| \lambda _i(G_S)-\frac{\sqrt{2}M}{n}\right| ,\qquad \text{ where } \quad M=\sum \limits _{v_i\in S}\frac{\sqrt{d_i(G_S)-1}}{d_i(G_S)} \end{aligned}$$

and $\lambda _1(G_S),\lambda _2(G_S), \ldots , \lambda _n(G_S)$ are the eigenvalues of $A_{ABC}(G_S)$. Let $\gamma _i(G_S)=\lambda _i(G_S)-\frac{\sqrt{2}M}{n}$, $1\le i\le n$ denotes the auxiliary eigenvalues of $A_{ABC}(G_S)$. Therefore $E_{ABC}(G_S)=\sum \limits _{i=1}^{n}\left| \gamma _i(G_S)\right| .$

In chemistry, topological indices have gained importance as tools for identifying correlations between the structural features of chemical compounds and their empirically determined physical and chemical properties (see^20–23^).

In the following sections of this article, we derive some bounds for *ABC* eigenvalues of a graph $G_S$ and $ABC(G_S)$ and $E_{ABC}(G_S)$. Also, we computed the *ABC* energy for complete graph, cocktail party graph and crown graph with self-loops. We also derived the characteristic polynomial of double star graph with self-loops. Additionally, we explore chemical importance of $ABC(G_S)$ is demonstrating that the ABC index is valuable for predicting the boiling point and molar refraction of certain chemical compounds, which are relevant in drug formulation and compare these indices.

## Bounds for ABC index and ABC energy of graph $G_S$

Let $G_S$ be a graph with self-loops obtained from simple graph *G* by attaching self-loops to each of the vertices belonging to *S*, then the eigenvalues of $A_{ABC}(G_S)$ satisfy,

1. $$\sum \limits _{i=1}^{n} \lambda _i(G_S) = \sqrt{2}M$$, where $$M=\sum \limits _{v_i\in S}\frac{\sqrt{d_i(G_S)-1}}{d_i(G_S)}$$.
2. $$\sum \limits _{i=1}^{n} \lambda ^2_i(G_S) = 2\left( \sum \limits _{v_iv_j\in E(G)}\frac{d_i(G_S)+d_j(G_S)-2}{d_i(G_S)d_j(G_S)}+\sum \limits _{v_i\in S}\frac{d_i(G_S)-1}{d^2_i(G_S)}\right)$$.

### Lemma 1

*Let* *G* *be a graph. If* $S\subseteq V(G)$, *then the auxiliary eigenvalues* $\gamma _1(G_S), \gamma _2(G_S), \ldots , \gamma _n(G_S)$ *of* $A_{ABC}(G_S)$ *satisfy*,

1. $$\sum \limits _{i=1}^{n}\gamma _i(G_S)=0$$.
2. $$\sum \limits _{i=1}^{n}\gamma _i^2(G_S)=2N$$, where $$N=\sum \limits _{v_iv_j\in E(G)}\frac{d_i(G_S)+d_j(G_S)-2}{d_i(G_S)d_j(G_S)}+\sum \limits _{v_i\in S}\frac{d_i(G_S)-1}{d^2_i(G_S)}-\frac{M^2}{n}$$.

### Proof

We have,

1. $$\sum \limits _{i=1}^{n}\gamma _i(G_S)=\sum \limits _{i=1}^{n}\left( \lambda _i(G_S)-\frac{\sqrt{2}M}{n}\right) =\sum \limits _{i=1}^{n}\lambda _i(G_S)-\sum \limits _{i=1}^{n}\frac{\sqrt{2}M}{n}=0$$.
2. Also,
  $$\begin{aligned} \sum \limits _{i=1}^{n}\gamma _i^2(G_S)=&\sum \limits _{i=1}^{n}\left( \lambda _i(G_S)-\frac{\sqrt{2}M}{n}\right) ^2\\ =&\sum \limits _{i=1}^{n}\lambda _i^2(G_S)+2\sum \limits _{i=1}^{n}\left( \frac{M}{n}\right) ^2-2\frac{\sqrt{2}M}{n}\sum \limits _{i=1}^{n}\lambda _i(G_S)\\ =&2\left( \sum \limits _{v_iv_j\in E(G)}\frac{d_i(G_S)+d_j(G_S)-2}{d_i(G_S)d_j(G_S)}+\sum \limits _{v_i\in S}\frac{d_i(G_S)-1}{d^2_i(G_S)}-\frac{M^2}{n}\right) =2N. \end{aligned}$$
  $$\square$$

### Lemma 2

^24^ *Let* *M* *be a matrix of order* *n* *and* $\alpha , \beta \in C$. *Then*, $\alpha$ *is an eigenvalue of* *M* *if and only if* $\alpha +\beta$ *is an eigenvalue of* $M+\beta I$.

### Lemma 3

^24^ *Let* $B \subset M_n$ *be Hermitian and let the eigenvalues of B be ordered as* $\zeta _1 \ge \zeta _2 \ge \cdots \ge \zeta _n$ *then* $\zeta _ny^*y \le y^*By \le \zeta _1 y^*y$,   $\forall y \in C^n$.

### Lemma 4

*Let* $\lambda _1(G_S) \ge \lambda _2(G_S) \ge \cdots \ge \lambda _n(G_S)$ *and* $\gamma _1(G_S)\ge \gamma _2(G_S)\ge \cdots \ge \gamma _n(G_S)$ *be the eigenvalues and auxiliary eigenvalues of* $A_{ABC}(G_S)$. *Then*,

1. $$\lambda _n(G_S) \le \frac{2ABC(G_S)-\sqrt{2}M}{n}\le \lambda _1(G_S)$$.
2. $$\gamma _n(G_S)\le \frac{2(ABC(G_S)-\sqrt{2}M)}{n}\le \gamma _1(G_S)$$.

### Proof

1. Let $\lambda _1(G_S) \ge \lambda _2(G_S) \ge \cdots \ge \lambda _n(G_S)$ be the eigenvalues of $A_{ABC}(G_S)$. Then for $y=1_n$ is a $n \times 1$ vector having all its entry 1 we get,
  $$\begin{aligned} y^TA_{ABC}(G_S)y =&\sum \limits _{i=1}^{n} \sum \limits _{j=1}^{n}[A_{ABC}(G_S)]_{ij}\\ =&2 \sum \limits _{v_i v_j \in E(G)} \sqrt{\frac{d_i(G_S)+d_j(G_S)-2}{d_i(G_S)d_j(G_S)}} + \sum \limits _{v_i \in S}\frac{\sqrt{2d_i(G_S)-2}}{d_i(G_S)}\\ =&2ABC(G_S) - \sqrt{2}M. \end{aligned}$$
  Therefore, by Lemma 3, $\lambda _n(G_S) \le \frac{2ABC(G_S)-\sqrt{2}M}{n}\le \lambda _1(G_S).$ The left equality holds if $A_{ABC}(G_S)y=\lambda _n(G_S)y$ and the right equality holds if $A_{ABC}(G_S)y=\lambda _1(G_S)y$.
2. Let $\gamma _1(G_S) \ge \gamma _2(G_S) \ge \cdots \ge \gamma _n(G_S)$ be the auxiliary eigenvalues of $A_{ABC}(G_S)$ then for $y=1_n$ we get,
  $$\begin{aligned} y^T\left( A_{ABC}(G_S)-\frac{\sqrt{2}M}{n}I\right) y=&\sum \limits _{i=1}^{n}\sum \limits _{j=1}^{n}\left[ A_{ABC}(G_S)-\frac{\sqrt{2}M}{n}I\right] _{ij}\\ =&2\sum \limits _{v_iv_j \in E(G)} \sqrt{\frac{d_i(G_S)+d_j(G_S)-2}{d_i(G_S)d_j(G_S)}}+ \sum \limits _{v_i\in S}\sqrt{\frac{2d_i(G_S)-2}{d_i(G_S)}}-\sqrt{2}M\\ =&2\left( ABC(G_S)-\sqrt{2}M\right) . \end{aligned}$$
  Therefore, by Lemma 2 and Lemma 3$\gamma _n\le \frac{2(ABC(G_S)-\sqrt{2}M)}{n}\le \gamma _1$. The left equality holds if $\left( A_{ABC}(G_S)-\frac{\sqrt{2}M}{n}I\right) y=\gamma _n(G_S)y$ and the right equality holds if $\left( A_{ABC}(G_S)-\frac{\sqrt{2}M}{n}I\right) y=\gamma _1(G_S)y$.

$$\square$$

### Lemma 5

^25^ *(Perron–Frobenious theorem) Let* $M\ge 0$ *be an irreducible matrix with spectral radius* $\alpha _\circ$. *Suppose* $l \in R ,$ and $y \ge 0 , y\ne 0$. *If* $My \le ly$ *then* $l\ge \alpha _\circ$.

### Theorem 1

*For* $n\ge 3$, *an upper bound for the* *ABC* *spectral radius of a graph* $G_S$ *is*

$$\begin{aligned} \lambda _1(G_S) \le (d_i+1){\frac{\sqrt{2n}}{d_i(G_S)}} \end{aligned}$$

*and equality holds if* $G_S \cong K_n$ *with all vertices having self-loops*.

### Proof

Let $x_i=\sqrt{d_i(G_S)}$. For a connected graph $G_S$, it is clear that for each vertex $v_i$, $d_i(G_S)\le n+1$. Consequently, for any vertex $v_i$ in $G_S$, it follows that

$$\begin{aligned} (ABC(G_S))_{x_i} \le&\sum \limits _{v_j \in N(v_i)} \sqrt{\frac{d_i(G_S)+d_j(G_S)-2}{d_i(G_S)d_j(G_S)}}{\sqrt{d_j(G_S)}}+ \sqrt{\frac{2d_i(G_S)-2}{d^2_i(G_S)}}{\sqrt{d_i(G_S)}}\\ =&\sum \limits _{v_j \in N(v_i)} \sqrt{\frac{d_i(G_S)+d_j(G_S)-2}{d_i(G_S)}}+ \sqrt{\frac{2d_i(G_S)-2}{d_i(G_S)}}\\ \le&d_i \sqrt{\frac{2n}{d_i(G_S)}}+\sqrt{\frac{2n}{d_i(G_S)}}\\ =&\left( (d_i+1){\frac{\sqrt{2n}}{d_i(G_S)}}\right) \sqrt{d_i(G_S)}. \end{aligned}$$

By Lemma 5, we have $\lambda _1(G_S) \le (d_i+1){\frac{\sqrt{2n}}{d_i(G_S)}}$. If equality holds, then it follows that for any two vertices $v_i$ and $v_j$, the equation $d_i+d_j=2n+2$ holds. Additionally, considering that $d_i\le n+1$, it follows that $d_i = n + 1$ for $i=1,2,\ldots ,n$. Thus, *G* is a complete graph in which every vertex includes a self-loop. $\square$

### Theorem 2

*Let* $G_S$ *be a graph obtained by adding a self-loop to each vertex in a graph* *G*. *Then*,

$$\begin{aligned} (m+n)\frac{\sqrt{2\delta +2}}{\Delta +2} \le ABC(G_S)\le (m+n)\frac{\sqrt{2\Delta +2}}{\delta +2}. \end{aligned}$$

*The upper and lower bound equalities occur for a regular graph*.

### Proof

Let $G_S$ be a graph obtained by adding a self-loop to every vertex of *G*. Then,

$$\begin{aligned} ABC(G_S)=&\sum \limits _{v_iv_j\in E(G)}\sqrt{\frac{d_i(G_S)+d_j(G_S)-2}{d_i(G_S)d_j(G_S)}}+\sum \limits _{i=1}^{n}\frac{\sqrt{2d_i(G_S)-2}}{d_i(G_S)}\\ =&\sum \limits _{v_iv_j\in E(G)}\sqrt{\frac{(d_i+2)+(d_j+2)-2}{(d_i+2)(d_j+2)}}+\sum \limits _{i=1}^{n}\frac{\sqrt{2(d_i+2)-2}}{(d_i+2)}\\ =&\sum \limits _{v_iv_j\in E(G)}\sqrt{\frac{d_i+d_j+2}{d_id_j+2(d_i+d_j)+4}}+\sum \limits _{i=1}^{n}\frac{\sqrt{2d_i+2}}{(d_i+2)}. \end{aligned}$$

But,

$$\begin{aligned} \sqrt{\frac{d_i+d_j+2}{d_id_j+2(d_i+d_j)+4}}\le \sqrt{\frac{2\Delta +2}{(\delta +2)^2}} \qquad \text {and} \qquad \frac{\sqrt{2d_i+2}}{(d_i+2)}\le \frac{\sqrt{2\Delta +2}}{\delta +2} \end{aligned}$$

1 $$\begin{aligned} ABC(G_S)&\le m\frac{\sqrt{2\Delta +2}}{\delta +2}+n\frac{\sqrt{2\Delta +2}}{\delta +2}\nonumber \\ ABC(G_S)&\le (m+n)\frac{\sqrt{2\Delta +2}}{\delta +2}. \end{aligned}$$

Similarly,

2 $$\begin{aligned} \sqrt{\frac{d_i+d_j+2}{d_id_j+2(d_i+d_j)+4}}\ge&\frac{\sqrt{2\delta +2}}{\Delta +2} \qquad \text {and}\qquad \frac{\sqrt{2d_i+2}}{(d_i+2)}\ge \frac{\sqrt{2\delta +2}}{\Delta +2}\nonumber \\ ABC(G_S)\ge (m+n)\frac{\sqrt{2\delta +2}}{\Delta +2}. \end{aligned}$$

From Eqs. 1 and 2

$$\begin{aligned} (m+n)\frac{\sqrt{2\delta +2}}{\Delta +2} \le ABC(G_S)\le (m+n)\frac{\sqrt{2\Delta +2}}{\delta +2}. \end{aligned}$$

The upper and lower bound equalities occur for a regular graph. $\square$

### Theorem 3

*Let* $G_S$ *be a graph obtained by attaching self-loops to each vertex of graph* *G*. Then, $ABC(G_S)\ge \sqrt{2N+n(n-1)D^{\frac{2}{n}}}$, where $D=\det \left( ABC(G_S)-\frac{\sqrt{2}M}{n}I_n\right)$. *Equality holds for* $n(K_1)_S$ *with all the vertices having self-loops*.

### Proof

By the arithmetic-geometric mean inequality,

$$\begin{aligned} \frac{1}{n(n-1)}\sum \limits _{i\ne j}\left| \gamma _i(G_S)\right| \left| \gamma _j(G_S)\right| \ge&\left( \prod \limits _{i\ne j}\left| \gamma _i(G_S)\right| \left| \gamma _j(G_S)\right| \right) ^{\frac{1}{n(n-1)}}\\ =&\left( \prod \limits _{i=1}^{n}\left| \gamma _i(G_S)\right| ^{2(n-1)}\right) ^{\frac{1}{n(n-1)}}\\ =&\left( \prod \limits _{i=1}^{n}\left| \gamma _i(G_S)\right| \right) ^{\frac{2}{n}}\\ =&\left( \prod \limits _{i=1}^{n}\left| \lambda _i(G_S)-\frac{\sqrt{2}M}{n}\right| \right) ^{\frac{2}{n}}\\ =&\left| \det \left( ABC(G_S)-\frac{\sqrt{2}M}{n}I_n\right) \right| ^{\frac{2}{n}}. \end{aligned}$$

Therefore,

3 $$\begin{aligned} \sum \limits _{i\ne j}\left| \gamma _i(G_S)\right| \left| \gamma _j(G_S)\right| \ge&n(n-1)D^{\frac{2}{n}}.\end{aligned}$$

Now consider,

4 $$\begin{aligned} \left( ABC(G_S)\right) ^2=&\left( \sum \limits _{i=1}^{n}\left| \gamma _i(G_S)\right| \right) ^2\nonumber \\ =&\sum \limits _{i=1}^{n}\gamma _i^2(G_S)+\sum \limits _{i\ne j}\left| \gamma _i(G_S)\right| \left| \gamma _j(G_S)\right| . \end{aligned}$$

Hence, by applying Lemma 1 and substituting Equation 3 into Equation 4, we obtain $ABC(G_S)\ge \sqrt{2N+n(n-1)D^{\frac{2}{n}}}$, where $D=\det \left( ABC(G_S)-\frac{\sqrt{2}M}{n}I_n\right)$. Equality holds for $n(K_1)_S$ with all the vertices having self-loops. $\square$

### Lemma 6

^26^ *Let* *M* *be any real symmetric matrix of order* *n*, $n\ge 2$. *Let C be any number satisfying* $\rho \left( M-\frac{k}{n}I\right) \ge C\ge \frac{||M-\frac{k}{n}I||_2}{\sqrt{n}}$. *Then*, $E_M\le C+\sqrt{(n-1)\left( ||M-\frac{k}{n}I||^2_2-C^2\right) }$.

### Theorem 4

*Let* $A_{ABC}(G_S)$ *be any real symmetric matrix of order* $n\ge 2$. *Then*,

$$\begin{aligned} E_{ABC}(G_S)\le \frac{2\left( ABC(G_S)-\sqrt{2}M\right) }{n}+\sqrt{(n-1)\left( 2N-\left( \frac{2\left( ABC(G_S)-\sqrt{2}M\right) }{n}\right) ^2\right) }. \end{aligned}$$

### Proof

Let $G_S$ be a connected graph of order $n\ge 2$ and $S\subseteq V(G)$. In order to prove the above bound we claim that,

$$\begin{aligned} \sqrt{\frac{2N}{n}}&\le \frac{2\left( ABC(G_S)-\sqrt{2}M\right) }{n}. \end{aligned}$$

Using Lemmas 1 and 2,

$$\begin{aligned} \sqrt{\frac{2N}{n}}&=\sqrt{\frac{\sum \limits _{i=1}^{n}\gamma ^2_i(G_S)}{n}}=\sqrt{\frac{\sum \limits _{i=1}^{n}\sum \limits _{j=1}^{n}\left[ A_{ABC}(G_S)-\frac{\sqrt{2}M}{n}I\right] }{n}}\\&\le \sqrt{\sum \limits _{i=1}^{n}\sum \limits _{j=1}^{n}\left( \left[ A_{ABC}(G_S)\right] _{ij}^2-\left[ \frac{\sqrt{2}M}{n}I\right] _{ij}^2\right) }\\&=\sqrt{\frac{\sum \limits _{i,j=1,i\ne j}^{n}\left[ A_{ABC}(G_S)\right] _{ij}^2+\sum \limits _{i=1}^{n}\left[ A_{ABC}(G_S)\right] _{ii}^2-\left( \frac{\sqrt{2}M}{n}\right) ^2n}{n}}\\&\le \frac{\sum \limits _{i,j=1,i\ne j}^{n}\left[ A_{ABC}(G_S)\right] _{ij}}{n}+\frac{\sum \limits _{i=1}^{n}\left[ A_{ABC}(G_S)\right] _{ii}}{n}-\frac{\sqrt{2}M}{n}\\&=\frac{2\left( ABC(G_S)-\sqrt{2}M\right) }{n}. \end{aligned}$$

Hence,

$$\begin{aligned} \sqrt{\frac{2N}{n}}&\le \frac{2\left( ABC(G_S)-\sqrt{2}M\right) }{n}. \end{aligned}$$

By Lemma 4,

$$\begin{aligned} \sqrt{\frac{2N}{n}}\le&\frac{2\left( ABC(G_S)-\sqrt{2}M\right) }{n}\le \gamma _1(G_S). \end{aligned}$$

Then by Lemma 6 we obtain,

$$\begin{aligned} E_{ABC}(G_S)\le \frac{2\left( ABC(G_S)-\sqrt{2}M\right) }{n}+\sqrt{(n-1)\left( 2N-\left( \frac{2\left( ABC(G_S)-\sqrt{2}M\right) }{n}\right) ^2\right) }. \end{aligned}$$

$$\square$$

### Theorem 5

*Let* $G_S$ *be a graph with* $\sigma$* self-loops. Then* $E_{ABC}(G_S)\ge 2\sqrt{N}$. *Equality holds for* $n(K_1)_S$ *with all the vertices having self-loops*.

### Proof

We know that

$$\begin{aligned} \sum \limits _{i=1}^{n}\gamma _i=&0=\left( \sum \limits _{i=1}^{n}\gamma _i\right) ^2=\sum \limits _{i=1}^{n}\gamma _i^2+2\sum \limits _{i\le j}\gamma _i\gamma _j.\\ \sum \limits _{i=1}^{n}\gamma _i^2=&-2\sum _{i\le j}\gamma _i\gamma _j \qquad \text {and}\qquad E_{ABC}(G_S)=\sum \limits _{i=1}^{n}|\gamma _i|. \end{aligned}$$

Now,

$$\begin{aligned} \left( E_{ABC}(G_S)\right) ^2=\left( \sum \limits _{i=1}^{n}|\gamma _i|\right) ^2 =&\sum \limits _{i=1}^{n}|\gamma _i|^2+2\sum \limits _{i\le j}|\gamma _i||\gamma _j|\\ \ge&\sum \limits _{i=1}^{n}|\gamma _i|^2+2\left| \sum \limits _{i\le j}\gamma _i\gamma _j\right| \\ =&\sum \limits _{i=1}^{n}|\gamma _i|^2+\left| \sum \limits _{i=1}^{n}-(\gamma _i)^2\right| \\ \ge&2\left| \sum \limits _{i=1}^{n}(\gamma _i)^2\right| \\ =&2(2N)\\ E_{ABC}(G_S)\ge&\sqrt{4N}=2\sqrt{N}. \end{aligned}$$

Equality holds for $n(K_1)_S$ with all the vertices having self-loops. $\square$

### Lemma 7

^27^ *Let* $p, p_1, \ldots , p_n, P$ *and* $q, q_1, \ldots , q_n, Q$ *be real numbers such that* $p\le p_i \le P$ *and* $q\le q_i \le Q$ *for all* $1\le i\le n$ *the following inequality is holds*.

$$\begin{aligned} \left| n\sum \limits _{i=1}^{n}p_iq_i-\sum \limits _{i=1}^{n}p_i\sum \limits _{i=1}^{n}q_i\right| \le \alpha (n)(P-p)(Q-q), \end{aligned}$$

*where* $\alpha (n)=n[\frac{n}{2}](1-\frac{1}{n}[\frac{n}{2}])$ *and* [*x*] *denote the integral part of real number x and equality holds* *if and only if* $p_1=p_2= \cdots  =p_n$ *and* $q_1=q_2= \cdots  =q_n$.

### Theorem 6

*Let* $\gamma _1(G_S), \gamma _2(G_S), \ldots , \gamma _n(G_S)$ *be the auxiliary eigenvalues of a graph* $G_S$. *Then*,

$$\begin{aligned} E_{ABC}(G_S)\ge \sqrt{n(2N)-\frac{n^2}{4}(|\gamma _1(G_S)|-\gamma _n(G_S)|)^2}. \end{aligned}$$

*Equality holds for* $n(K_1)_S$ *with all the vertices having self-loops*.

### Proof

Let $|\gamma _1(G_S)|\ge |\gamma _2(G_S)|\ge \cdots \ge |\gamma _n(G_S)|$. By substituting $a_i=b_i=|\gamma _i(G_S)|$, $a=b=|\gamma _n(G_S)|$ and $A=B=|\gamma _1(G_S)|$ in Lemma 7 and noting that $\alpha (n)\le \frac{n^2}{4}$. We get,

$$\begin{aligned} \left| n\sum \limits _{i=1}^{n}|\gamma _i(G_S)|^2-\left( \sum _{i=1}^{n}|\gamma _i(G_S)|\right) ^2\right| \le \frac{n^2}{4}\left( |\gamma _1(G_S)|-|\gamma _n(G_S)|\right) ^2. \end{aligned}$$

Since,

$$\begin{aligned} \sum \limits _{i=1}^{n}|\gamma _i(G_S)|=E_{ABC}(G_S)\qquad \text{ and }\qquad \sum \limits _{i=1}^{n}|\gamma _i^2(G_S)|=2N. \end{aligned}$$

We have,

$$\begin{aligned} n(2N)-(E_{ABC}(G_S))^2\le \frac{n^2}{4}\left( |\gamma _1(G_S)|-|\gamma _n(G_S)|\right) ^2.\end{aligned}$$

Therefore,

$$\begin{aligned} E_{ABC}(G_S)\ge \sqrt{n(2N)-\frac{n^2}{4}(|\gamma _1(G_S)|-|\gamma _n(G_S)|)^2}. \end{aligned}$$

Equality holds for $n(K_1)_S$ with all the vertices having self-loops. $\square$

### Lemma 8

^27^ *Let* $u_i\ne 0, v_i, r$ *and* *R* *be real numbers satisfying* $ru_i \le qv_i \le Ru_i$. *Then following inequality holds*,

$$\begin{aligned} \sum _{i=1}^{n}v_i^2+rR\sum _{i=1}^{n}u_i\le (r+R)\sum _{i=1}^{n}u_iv_i. \end{aligned}$$

*Equality holds if* $rx_i=y_i=Rx_i$ *for at least one* *i*.

### Theorem 7

*Let* $\gamma _1(G_S), \gamma _2(G_S), \ldots , \gamma _n(G_S)$ *be the auxiliary eigenvalues of a graph* $G_S$ *containing* $\sigma$ *self-loops. Then*,

$$\begin{aligned} E_{ABC}(G_S)\ge \frac{2N+n|\gamma _1(G_S)||\gamma _n(G_S)|}{|\gamma _1(G_S)|+|\gamma _n(G_S)|}. \end{aligned}$$

*Equality holds for* $n(K_1)_S$ *with all the vertices having self-loops*.

### Proof

Let $|\gamma _1(G_S)|\ge |\gamma _2(G_S)\ge \cdots \ge |\gamma _n(G_S)|$. By substituting $a_i=1, b_i=|\gamma _i(G_S)|$, $r=|\gamma _n(G_S)|$ and $R=|\gamma _1(G_S)|$ in Lemma 8. we get,

$$\begin{aligned} \sum \limits _{i=1}^{n}|\gamma _i(G_S)|^2+|\gamma _1(G_S)||\gamma _n(G_S)|\sum \limits _{i=1}^{n}1\le \left( |\gamma _1(G_S)|+|\gamma _n(G_S)|\right) \sum \limits _{i=1}^{n}|\gamma _i(G_S)|. \end{aligned}$$

Since,

$$\begin{aligned} \sum \limits _{i=1}^{n}|\gamma _i(G_S)|=E_{ABC}(G_S) \qquad \text{ and }\qquad \sum \limits _{i=1}^{n}|\gamma _i^2(G_S)|=2N. \end{aligned}$$

We have,

$$\begin{aligned} 2N+n|\gamma _1(G_S)||\gamma _n(G_S)|\le (|\gamma _1(G_S)|+|\gamma _n(G_S)|)E_{ABC}(G_S).\end{aligned}$$

Therefore,

$$\begin{aligned} E_{ABC}(G_S)\ge \frac{2N+n|\gamma _1(G_S)||\gamma _n(G_S)|}{|\gamma _1(G_S)|+|\gamma _n(G_S)|}. \end{aligned}$$

Equality holds for $n(K_1)_S$ with all the vertices having self-loops. $\square$

### Theorem 8

*Let G be a graph with first Zagreb index* $M_1(G)$ *and* $G_S$ *be a graph with self-loops in which all the vertices having a self-loops. Then* $E_{ABC}(G_S)\ge \frac{2\sqrt{M_1(G)+2m+\sigma (\delta +1)-\frac{M^2}{n}(\Delta +1)^2}}{\Delta +2}$.

### Proof

Let

$$\begin{aligned} (E_{ABC}(G_S))^2&=\left( \sum \limits _{i=1}^{n}|\gamma _i(G_S)|\right) ^2\\&=\sum \limits _{i=1}^{n}|\gamma _i|^2+2\sum \limits _{1\le i<j\le n}^{n}|\gamma _i(G_S)||\gamma _j(G_S)|\\&\ge \sum \limits _{i=1}^{n}\gamma _i^2+2\left| \sum \limits _{i=1}^{n}\gamma _i\gamma _j\right| \\&=2\sum \limits _{i=1}^{n}\gamma _i^2\\&=4\left( \sum \limits _{v_iv_j\in E(G)}\frac{d_i(G_S)+d_j(G_S)-2}{d_i(G_S)d_j(G_S)}+\sum \limits _{v_i\in S}\frac{d_i(G_S)-1}{d^2_i(G_S)}-\frac{M^2}{n}\right) \\&=4\sum \limits _{v_iv_j\in E(G)}\frac{d_i+d_j+2}{(d_i+2)(d_j+2)}+4\sum \limits _{v_i\in S}\frac{d_i+1}{(d_i+2)^2}-\frac{M^2}{n}\\&\ge \frac{4M_1(G)}{(\Delta +2)^2}+\frac{8m}{(\Delta +2)^2}+\frac{4\sigma (\delta +1)}{(\Delta +2)^2}-\frac{M^2}{n}\\ E_{ABC}(G_S)&\ge \frac{2\sqrt{M_1(G)+2m+\sigma (\delta +1)-\frac{M^2}{n}(\Delta +1)^2}}{\Delta +2}. \end{aligned}$$

$$\square$$

## ABC energy of a graph with self-loops

### Theorem 9

*For a complete graph* $(K_n)_S$ *with* $\sigma$ *self-loops*,

$$\begin{aligned} E_{ABC}(K_n)_S=(n-\sigma -1)\left( \frac{\sqrt{2n-4}}{n-1}\right) +(n-2)\left( \frac{\sigma \sqrt{2n}}{n(n+1)}\right) +\sqrt{\left( \frac{\sigma \sqrt{2n}}{n+1}-(n-\sigma -1)\frac{\sqrt{2n-4}}{n-1}\right) ^2+8\left( \frac{n\sigma -\sigma ^2}{n+1}\right) }. \end{aligned}$$

### Proof

Let $(K_n)_S$ be a complete graph with $\sigma$ self-loops. Then

$$A_{ABC}(K_n)_S = \begin{bmatrix} \sqrt{\frac{2n}{(n+1)^2}}J_{\sigma \times \sigma } & \sqrt{\frac{2n-2}{n^2-1}}J_{\sigma \times (n-\sigma )} \\ \sqrt{\frac{2n-2}{n^2-1}}J_{(n-\sigma )\times \sigma } & \sqrt{\frac{2n-4}{(n-1)^2}}(J-I)_{(n-\sigma )\times (n-\sigma )}\\ \end{bmatrix}$$

Consider $$\det (\lambda I-A_{ABC}(K_n)_S)$$.

Step 1: Replace $C_i$ by $C_i-C_{i+1}$, where $1\le i\le \sigma -1$, $\sigma +1\le i\le n-1$. Then it simplifies to the new determinant, that is $\det (F)$.
Step 2: In $\det (F)$, replace $R_i$ by $R_i+R_{i-1}$, where $2\le i \le \sigma$, $\sigma +2 \le i \le n$. Then we conclude $\det (\lambda I-A_{ABC}(K_n)_S)$ is of the form
  $$\begin{aligned} \det (\lambda I-A_{ABC}(K_n)_S)=&(-\lambda (K_n)_S)^{\sigma -1}\left[ -\left( \sqrt{\frac{2n-4}{(n-1)^2}}+\lambda (K_n)_S\right) \right] ^{n-\sigma -1} \begin{vmatrix} \sigma \sqrt{\frac{2n}{(n+1)^2}}-\lambda (K_n)_S&\sigma \sqrt{\frac{2n-2}{n^2-1}} \\ (n-\sigma )\sqrt{\frac{2n-2}{n^2-1}}&(n-1-\sigma )\sqrt{\frac{2n-4}{(n-1)^2}}-\lambda (K_n)_S\\ \end{vmatrix} \end{aligned}$$

Then, the *ABC* spectrum of complete graph with self-loops is given by,

$$\begin{pmatrix} 0 & -\sqrt{\frac{2n-4}{(n-1)^2}} & P & R \\ \sigma -1 & n-\sigma -1 & 1 & 1\\ \end{pmatrix}$$

where,

$$\begin{aligned} P=\frac{\frac{\sigma \sqrt{2n}}{n+1}+(n-1-\sigma )\frac{\sqrt{2n-4}}{n-1}+\sqrt{\left( \frac{\sigma \sqrt{2n}}{n+1}-(n-\sigma -1)\frac{\sqrt{2n-4}}{n-1}\right) ^2+8\left( \frac{n\sigma -\sigma ^2}{n+1}\right) }}{2}\text {and} \\ R=\frac{\frac{\sigma \sqrt{2n}}{n+1}+(n-1-\sigma )\frac{\sqrt{2n-4}}{n-1}-\sqrt{\left( \frac{\sigma \sqrt{2n}}{n+1}-(n-\sigma -1)\frac{\sqrt{2n-4}}{n-1}\right) ^2+8\left( \frac{n\sigma -\sigma ^2}{n+1}\right) }}{2}. \end{aligned}$$

Therefore, the *ABC* energy of complete graph with self-loops is given by,

$$\begin{aligned} E_{ABC}(K_n)_S&=(\sigma -1)\left| \frac{-\sigma \sqrt{2n}}{n(n+1)}\right| +(n-\sigma -1)\left| \frac{-\sqrt{2n-4}}{n-1}-\frac{\sigma \sqrt{2n}}{n(n+1)}\right| +\left| P-\frac{\sigma \sqrt{2n}}{n(n+1)}\right| +\left| R-\frac{\sigma \sqrt{2n}}{n(n+1)}\right| . \end{aligned}$$

Further simplification will result in,

$$\begin{aligned} E_{ABC}(K_n)_S=(n-\sigma -1)\left( \frac{\sqrt{2n-4}}{n-1}\right) +(n-2)\frac{\sigma \sqrt{2n}}{n(n+1)}+&\sqrt{\left( \frac{\sigma \sqrt{2n}}{n+1}-(n-\sigma -1)\frac{\sqrt{2n-4}}{n-1}\right) ^2+8\left( \frac{n\sigma -\sigma ^2}{n+1}\right) }. \end{aligned}$$

$$\square$$

### Definition 1

^28^ The cocktail party graph, denoted by $k_{n\times 2}$, is a graph having vertex set $V(G)=\prod \limits _{i=1}^{n}u_i,v_i$ and edge set $E(G)=\{u_iu_j,v_iv_j,u_iv_j,v_iu_j:1\le i,j\le n\}$. In other words, the cocktail part graph of order 2*n*, is the graph consisting of two paired vertices in which all vertices but the paired ones are connected with a graph edge. This graph is also called complete $n-$ partite graph.

### Lemma 9

^29^ *Let* $$A = \begin{bmatrix} A_\circ & A_1 \\ A_1 & A_\circ \\ \end{bmatrix}$$ *be a block symmetric matrix of order 2. Then the eigenvalues of A are the eigenvalues of the matrices *$A_\circ + A_1$ *and* $A_\circ -A_1$.

### Theorem 10

*Let* $(K_{2n\times 2})_S$ *be a cocktail party graph with* $\sigma =\sigma _1+\sigma _2$ *self-loops having vertex set* $V=\{v_i, u_i|1\le i \le 2n\}$, *and the partition* $P=\{V_1, V_2\}$, *such that* $V_1$ *and* $V_2$ *contains* $v_i$ and $u_i$ *vertices respectively. The first* $\sigma _1$ *vertices in* $V_1$ *and first* $\sigma _2$ *vertices in* $V_2$ *have self-loops, where* $\sigma _1=\sigma _2$ *and* $\sigma _1,\sigma _2\ge 2$. *Then*

$$\begin{aligned} E_{ABC}(K_{2n\times 2})_S=&(2n-\sigma _1-1)\left( \frac{\sqrt{8n-6}}{2n-1}\right) +\left( \frac{8n\sigma _1-n-4\sigma _1}{4n^2}\right) \sqrt{8n-2}+\\ &\sqrt{\left( (2\sigma _1-1)\frac{\sqrt{8n-2}}{4n}-2(2n-\sigma _1-1)\frac{\sqrt{8n-6}}{4n-2}\right) ^2+16\sigma _1 (2n-\sigma _1)\left( \frac{1}{2n}\right) }. \end{aligned}$$

### Proof

Let $(K_{2n\times 2})_S$ be a cocktail party graph with $\sigma =\sigma _1+\sigma _2$ self-loops having vertex set $V=\{v_i, u_i|1\le i \le 2n\}$, and the partition $P=\{V_1, V_2\}$, such that $V_1$ and $V_2$ contains $v_i$ and $u_i$ vertices respectively. The first $\sigma _1$ vertices in $V_1$ and first $\sigma _2$ vertices in $V_2$ have self-loops, where $\sigma _1=\sigma _2$ and $\sigma _1,\sigma _2\ge 2$. Then $A_{ABC}(K_{2n\times 2})_S=$

$$\begin{bmatrix} \sqrt{\frac{8n-2}{(4n)^2}}J_{\sigma _1} & \sqrt{\frac{1}{2n}}J_{\sigma _1\times (2n-\sigma _1)} & \sqrt{\frac{8n-2}{(4n)^2}}(J-I)_{\sigma _1\times \sigma _2} & \sqrt{\frac{1}{2n}}J_{\sigma _1\times (2n-\sigma _2)} \\ \sqrt{\frac{1}{2n}}J_{(2n-\sigma _1)\times \sigma _1} & \sqrt{\frac{8n-6}{(4n-2)^2}}(J-I)_{(2n-\sigma _1)} & \sqrt{\frac{1}{2n}}J_{(2n-\sigma _1)\times \sigma _2} & \sqrt{\frac{8n-6}{(4n-2)^2}}(J-I)_{(2n-\sigma _1)\times (2n-\sigma _2)}\\ \sqrt{\frac{8n-2}{(4n)^2}}(J-I)_{\sigma _2\times \sigma _1} & \sqrt{\frac{1}{2n}}J_{\sigma _2\times (2n-\sigma _1)} & \sqrt{\frac{8n-2}{(4n)^2}}J_{\sigma _2} & \sqrt{\frac{1}{2n}}J_{\sigma _2\times (2n-\sigma _2)} \\ \sqrt{\frac{1}{2n}}J_{(2n-\sigma _2)\times \sigma _1} & \sqrt{\frac{8n-6}{(4n-2)^2}}(J-I)_{(2n-\sigma _2)\times (2n-\sigma _1)} & \sqrt{\frac{1}{2n}}J_{(2n-\sigma _2)\times \sigma _2} & \sqrt{\frac{8n-6}{(4n-2)^2}}J_{(2n-\sigma _2)} \\  \end{bmatrix}$$

Consider $$\det (\lambda I-A_{ABC}(K_{2n\times 2})_S)$$.

Step 1: Replace $C_i$ by $C_i-C_{i+1}$, where $1 \le i \le \sigma _1-1, \sigma _1+1 \le i \le 2n-1,1 \le i \le \sigma _2-1$ and $\sigma _2+1 \le i \le 2n-1$. Then it simplifies to the new determinant, that is $\det (F)$.
Step 2: In $\det (F)$, replace $R_{i+1}$ by $R_{i+1}-R_i$, where $1 \le i \le \sigma _1-1, \sigma _1+1 \le i \le 2n-1, 1 \le i \le \sigma _2-1$ and $\sigma _2+1 \le i \le 2n-1$.

Hence, from Lemma 9, we get the *ABC* spectrum of $(K_{2n\times 2})_S$ is

$$\begin{pmatrix} -\frac{\sqrt{8n-2}}{4n} & -2\frac{\sqrt{8n-6}}{4n-2} & \frac{\sqrt{8n-2}}{4n} & 0 & P & R \\ \sigma _1-1 & 2n-\sigma _1-1 & \sigma _1 & 2n-\sigma _1 & 1 & 1\\ \end{pmatrix}$$

where,

$$\begin{aligned} P=&\frac{(2\sigma _1-1)\frac{\sqrt{8n-2}}{4n}+(4n-2\sigma _1-2)\frac{\sqrt{8n-6}}{4n-2}}{2}+\frac{\sqrt{\left( (2\sigma _1-1)\frac{\sqrt{8n-2}}{4n}-2(2n-\sigma _1 -1)\frac{\sqrt{8n-6}}{4n-2}\right) ^2+16\sigma _1 (2n-\sigma _1)\left( \frac{1}{2n}\right) }}{2}\text {and} \end{aligned}$$

$$\begin{aligned} R=&\frac{(2\sigma _1-1)\frac{\sqrt{8n-2}}{4n}+(4n-2\sigma _1-2)\frac{\sqrt{8n-6}}{4n-2}}{2}-\frac{\sqrt{\left( (2\sigma _1-1)\frac{\sqrt{8n-2}}{4n}-2(2n-\sigma _1 -1)\frac{\sqrt{8n-6}}{4n-2}\right) ^2+16\sigma _1 (2n-\sigma _1)\left( \frac{1}{2n}\right) }}{2}. \end{aligned}$$

Then, the *ABC* energy of cocktail party graph $(K_{2n\times 2})_S$ with self-loops is given by,

$$\begin{aligned} E_{ABC}(K_{2n\times 2})_S=&(2n-\sigma _1-1)\left( \frac{\sqrt{8n-6}}{2n-1}\right) +\left( \frac{8n\sigma _1-n-4\sigma _1}{4n^2}\right) \sqrt{8n-2}+\\ &\sqrt{\left( (2\sigma _1-1)\frac{\sqrt{8n-2}}{4n}-2(2n-\sigma _1-1)\frac{\sqrt{8n-6}}{4n-2}\right) ^2+16\sigma _1 (2n-\sigma _1)\left( \frac{1}{2n}\right) }. \end{aligned}$$

$$\square$$

### Corollary 1

Let $(K_{(2n+1)\times 2})_S$ be a cocktail party graph with $\sigma =\sigma _1+\sigma _2$ self-loops having vertex set $V=\{v_i, u_i|1\le i \le 2n+1\}$, and the partition $P=\{V_1, V_2\}$, such that $V_1$ and $V_2$ contains $v_i$ and $u_i$ vertices respectively. The first $\sigma _1$ vertices in $V_1$ and first $\sigma _2$ vertices in $V_2$ have self-loops, where $\sigma _1=\sigma _2$ and $\sigma _1,\sigma _2\ge 2$. Then

$$\begin{aligned} E_{ABC}\left( K_{(2n+1)\times 2}\right) _S=&(8\sigma _1-1)\frac{8n-2}{4n+2}+(4n-2\sigma _1)\frac{8n-2}{4n}+\\&\sqrt{\left( (2\sigma _1-1)\frac{\sqrt{8n+2}}{4n+2}-2(2n-\sigma _1)\frac{\sqrt{8n-2}}{4n}\right) ^2+16\sigma _1 (2n+1-\sigma _1)\left( \frac{2}{4n+2}\right) }. \end{aligned}$$

The proof of this corollary is similar to the one above; therefore, we will omit it.

### Definition 2

^28^ The crown graph $S_n^0$ for an integer $n\ge 3$ is the graph with vertex set $\{u_1,u_2,\ldots ,u_n,v_1,v_2,\ldots ,v_n\}$ and edge set $\{u_iv_j:1\le i,j\le n,i\ne j\}$. It is equivalent to the complete bipartite graph $K_{n,n}$ with horizontal edges $u_iv_i$ removed.

### Theorem 11

*Let* $(S_{n}^{0})_S$ *be a crown graph with* $\sigma =\sigma _1+\sigma _2$ *self-loops having vertex set* $V=\{v_i, u_i|1\le i \le n\}$, *and the partition* $P=\{V_1, V_2\}$, *such that* $V_1$ *and* $V_2$ *contains* $v_i$ *and* $u_i$ *vertices respectively. The first* $\sigma _1$ *vertices in* $V_1$ *and first* $\sigma _2$ *vertices in* $V_2$ *have self-loops, where* $\sigma _1=\sigma _2$ *and* $\sigma _1,\sigma _2\ge 2$. Then

$$\begin{aligned} E_{ABC}(S_{n}^{0})_S=&(\sigma _1-1)\frac{2\sqrt{2n}}{n+1}+2(n-\sigma _1-1)\frac{\sqrt{2n-4}}{n-1}+\sqrt{\left( \sigma _1\frac{\sqrt{2n}}{n+1}-(n-\sigma _1-1)\frac{\sqrt{2n-4}}{n-1}\right) ^2+4\sigma _1(n-\sigma _1)\left( \frac{2n-2}{(n+1)(n-1)}\right) }\\ &+\sqrt{\left( (-\sigma _1+2)\frac{\sqrt{2n}}{n+1}-(n-\sigma _1-1)\frac{\sqrt{2n-4}}{n-1}\right) ^2+4\sigma _1(n-\sigma _1)\left( \frac{2n-2}{(n+1)(n-1)}\right) }. \end{aligned}$$

### Proof

Let $(S_{n}^{0})_S$ be a crown graph with $\sigma =\sigma _1+\sigma _2$ self-loops having vertex set $V=\{v_i, u_i|1\le i \le n\}$, and the partition $P=\{V_1, V_2\}$, such that $V_1$ and $V_2$ contains $v_i$ and $u_i$ vertices respectively. The first $\sigma _1$ vertices in $V_1$ and first $\sigma _2$ vertices in $V_2$ have self-loops, where $\sigma _1=\sigma _2$ and $\sigma _1,\sigma _2\ge 2$. Then $A_{ABC}(S_{n}^{0})_S=$

$$\begin{bmatrix} \sqrt{\frac{2n}{(n+1)^2}}J_{\sigma _1} & 0_{\sigma _1\times (n-\sigma _1)} & \sqrt{\frac{2n}{(n+1)^2}}(J-I)_{\sigma _1\times \sigma _2} & \sqrt{\frac{2n-2}{(n+1)(n-1)}}J_{\sigma _1\times (n-\sigma _2)} \\ 0_{(n-\sigma _1)\times \sigma _1} & 0_{(n-\sigma _1)} & \sqrt{\frac{2n-2}{(n+1)(n-1)}}J_{(n-\sigma _1)\times \sigma _2} & \sqrt{\frac{2n-4}{(n-1)^2}}(J-I)_{(n-\sigma _1)\times (n-\sigma _2)}\\ \sqrt{\frac{2n}{(n+1)^2}}(J-I)_{\sigma _2\times \sigma _1} & \sqrt{\frac{2n-2}{(n+1)(n-1)}}J_{\sigma _2\times (n-\sigma _1)} & \sqrt{\frac{2n}{(n+1)^2}}J_{\sigma _2} & 0_{\sigma _2\times (n-\sigma _2)} \\ \sqrt{\frac{2n-2}{(n+1)(n-1)}}J_{(n-\sigma _2)\times \sigma _1} & \sqrt{\frac{2n-4}{(n-1)^2}}(J-I)_{(n-\sigma _2)\times (n-\sigma _1)} & 0_{(n-\sigma _2)\times \sigma _2} & 0_{(n-\sigma _2)} \\ \end{bmatrix}$$

Consider $$\det (\lambda I-A_{ABC}(S_{n}^{0})_S)$$.

Step 1: Replace $C_i$ by $C_i-C_{i+1}$, where $1 \le i \le \sigma _1-1, \sigma _1+1 \le i \le n-1,1 \le i \le \sigma _2-1$ and $\sigma _2+1 \le i \le n-1$. Then it simplifies to the new determinant, that is $\det (F)$.
Step 2: In $\det (F)$, replace $R_{i+1}$ by $R_{i+1}-R_i$, where $1 \le i \le \sigma _1-1, \sigma _1+1 \le i \le n-1, 1 \le i \le \sigma _2-1$ and $\sigma _2+1 \le i \le n-1$. Hence, from Lemma 9, we get the *ABC* spectrum of $(S_{n}^{0})_S$ is
  $$\begin{pmatrix} -\frac{\sqrt{2n-4}}{n-1} & 0& \frac{\sqrt{2n-4}}{n-1} & 2\frac{\sqrt{2n}}{n+1} & P & R& X& Y \\ n-\sigma _1-1 & \sigma _1-1 & n-\sigma _1-1 & \sigma _1-1 & 1 & 1& 1& 1\\ \end{pmatrix}$$

where,

$$\begin{aligned} P=&\frac{\sigma _1\frac{\sqrt{2n}}{n+1}+(n-\sigma _1-1)\frac{\sqrt{2n-4}}{n-1}}{2}+\frac{\sqrt{\left( \sigma _1\frac{\sqrt{2n}}{n+1}-(n-\sigma _1 -1)\frac{\sqrt{2n-4}}{n-1}\right) ^2+4\sigma _1 (n-\sigma _1)\left( \frac{2n-2}{(n+1)(n-1)}\right) }}{2}, \end{aligned}$$

$$\begin{aligned} R=&\frac{\sigma _1\frac{\sqrt{2n}}{n+1}+(n-\sigma _1-1)\frac{\sqrt{2n-4}}{n-1}}{2}-\frac{\sqrt{\left( \sigma _1\frac{\sqrt{2n}}{n+1}-(n-\sigma _1 -1)\frac{\sqrt{2n-4}}{n-1}\right) ^2+4\sigma _1 (n-\sigma _1)\left( \frac{2n-2}{(n+1)(n-1)}\right) }}{2}, \end{aligned}$$

$$\begin{aligned} X=&\frac{(-\sigma _1+2)\frac{\sqrt{2n}}{n+1}-(n-\sigma _1-1)\frac{\sqrt{2n-4}}{n-1}}{2}+\frac{\sqrt{\left( (-\sigma _1+2)\frac{\sqrt{2n}}{n+1}-(n-\sigma _1 -1)\frac{\sqrt{2n-4}}{n-1}\right) ^2+4\sigma _1 (n-\sigma _1)\left( \frac{2n-2}{(n+1)(n-1)}\right) }}{2}\text {and} \end{aligned}$$

$$\begin{aligned} Y=&\frac{(-\sigma _1+2)\frac{\sqrt{2n}}{n+1}-(n-\sigma _1-1)\frac{\sqrt{2n-4}}{n-1}}{2}-\frac{\sqrt{\left( (-\sigma _1+2)\frac{\sqrt{2n}}{n+1}-(n-\sigma _1 -1)\frac{\sqrt{2n-4}}{n-1}\right) ^2+4\sigma _1 (n-\sigma _1)\left( \frac{2n-2}{(n+1)(n-1)}\right) }}{2}. \end{aligned}$$

Then, the *ABC* energy of crown graph $(S_{n}^{0})_S$ with self-loops is given by,

$$\begin{aligned} E_{ABC}(S_{n}^{0})_S=&(\sigma _1-1)\frac{2\sqrt{2n}}{n+1}+2(n-\sigma _1-1)\frac{\sqrt{2n-4}}{n-1}+\sqrt{\left( \sigma _1\frac{\sqrt{2n}}{n+1}-(n-\sigma _1-1)\frac{\sqrt{2n-4}}{n-1}\right) ^2+4\sigma _1(n-\sigma _1)\left( \frac{2n-2}{(n+1)(n-1)}\right) }\\ &+\sqrt{\left( (-\sigma _1+2)\frac{\sqrt{2n}}{n+1}-(n-\sigma _1-1)\frac{\sqrt{2n-4}}{n-1}\right) ^2+4\sigma _1(n-\sigma _1)\left( \frac{2n-2}{(n+1)(n-1)}\right) }. \end{aligned}$$

$$\square$$

### Definition 3

^30^ The double star *S*(*m*, *n*) is a tree of diameter three such that there are $m-1$ pendent edges on one end of the path and $n-1$ pendent edges on the other end.

### Theorem 12

*Let* $(S_{m,n})_S$ *be a double star graph with* $\sigma =\sigma _1+\sigma _2$ *self-loops having a vertex set* $V=\{v_i, u_j|1\le i\le m, 1\le j\le n\}$, *and partition* $P=\{V_1, V_2\}$, *such that* $V_1$ *and* $V_2$ *contains* $v_i$ and $u_j$ *vertices respectively, where* $\sigma _1=\sigma _2$. *Then the characteristic polynomial is* $\left( \lambda (S_{m,n})_S-\frac{2}{3}\right) ^{2\sigma _1-2}\left( \lambda (S_{m,n})_S)\right) ^{m+n-2\sigma _1-4}Q(\lambda (S_{m,n})_S)$, where

$$\begin{aligned}& Q(\lambda (S_{m,n})_S)=\frac{1}{9mn}[ 9mn\lambda ^6(S_{m,n})_S-12mn\lambda ^5(S_{m,n})_S\\ &+( 12\sigma _1mn-12\sigma _1m-12\sigma _1n-9m^2n-9mn^2+40mn-18m-18n+18) \\ &\lambda ^4(S_{m,n})_S+( -20\sigma _1mn+14\sigma _1m+14\sigma _1n+12m^2n+12mn^2-48mn+24m+24n-24) \\ &\lambda ^3(S_{m,n})_S+( 4\sigma _1^2mn+16\sigma _1^2-6\sigma _1m^2n+12\sigma _1m^2-6\sigma _1mn^2+32\sigma _1mn-34\sigma _1m \\&+12\sigma _1n^2-34\sigma _1n+24\sigma _1+9m^2n^2-22m^2n+9m^2-22mn^2+52mn-26m+9n^2-26n+17) \\ & \lambda ^2(S_{m,n})_S+( -8\sigma _1^2mn-8\sigma _1^2m-8\sigma _1^2n-16\sigma _1^2+10\sigma _1m^2n-14\sigma _1m^2 \\& +10\sigma _1mn^2-40\sigma _1mn+38\sigma _1m-14\sigma _1n^2+38\sigma _1n-28\sigma _1-12m^2n^2+24m^2n-12m^2+24mn^2-48mn+24m+24n-12) \\ & \lambda (S_{m,n})_S+( 4\sigma _1^2mn-4\sigma _1^2m-4\sigma _1^2n+4\sigma _1^2-4\sigma _1m^2n+4\sigma _1m^2-4\sigma _1mn^2+16\sigma _1mn-12\sigma _1m+4\sigma _1n^2-12\sigma _1n+8\sigma _1 \\& +4m^2n^2-8m^2n+4m^2-8mn^2+16mn+4n^2-8n+4) ] . \end{aligned}$$

### Proof

Let $(S_{m,n})_S$ be a double star graph with $\sigma =\sigma _1+\sigma _2$ self-loops having a vertex set $V=\{v_i, u_j|1\le i\le m, 1\le j\le n\}$, and partition $P=\{V_1, V_2\}$, such that $V_1$ and $V_2$ contains $v_i$ and $u_j$ vertices respectively, where $\sigma _1=\sigma _2$. Then $A_{ABC}(S_{m,n})_S=$

$$\begin{bmatrix} \frac{2}{3}I_{\sigma _1} & 0_{\sigma _1\times (m-\sigma _1-1)} & \sqrt{\frac{m+1}{3m}}J_{\sigma _1\times 1} & 0_{\sigma _1\times \sigma _2}& 0_{\sigma _1\times (n-\sigma _2-1)}& 0_{\sigma _1\times 1} \\ 0_{(m-\sigma _1-1)\times \sigma _1} & 0 {(m-\sigma _1-1)} & \sqrt{\frac{m-1}{m}}J_{(m-\sigma _1-1)\times 1}& 0_{(m-\sigma _1-1)\times \sigma _2}& 0_{(m-\sigma _1-1)\times (n-\sigma _2-1)}& 0_{(m-\sigma _1-1)\times 1}\\ \sqrt{\frac{m+1}{3m}}J_{1\times \sigma _1} & \sqrt{\frac{m-1}{m}}J_{1\times (m-\sigma _1-1)} & 0_{1\times 1} & 0_{1\times \sigma _2}& 0_{1\times (n-\sigma _2-1)}& \sqrt{\frac{m+n-2}{mn}}J_{1\times 1} \\ 0_{\sigma _2\times \sigma _1}& 0_{\sigma _2\times (m-\sigma _1-1)}& 0_{\sigma _2\times 1}& \frac{2}{3}I_{\sigma _2} & 0_{\sigma _2\times (n-\sigma _2-1)} & \sqrt{\frac{n+1}{3n}}J_{\sigma _2\times 1}\\ 0_{(n-\sigma _2-1)\times \sigma _1}& 0_{(n-\sigma _2-1)\times (m-\sigma _1-1)}& 0_{(n-\sigma _2-1)\times 1}& 0_{(n-\sigma _2-1)\times \sigma _2} & 0 {(n-\sigma _2-1)} & \sqrt{\frac{n-1}{n}}J_{(n-\sigma _2-1)\times 1}\\ 0_{1\times \sigma _1}& 0_{1\times (m-\sigma _1-1)}& \sqrt{\frac{m+n-2}{mn}}J_{1\times 1}& \sqrt{\frac{n+1}{3n}}J_{1\times \sigma _2} & \sqrt{\frac{n-1}{n}}J_{1\times (n-\sigma _2-1)} & 0_{1\times 1} \\ \end{bmatrix}$$

Consider $$\det (\lambda I-A_{ABC}(S_{m,n})_S)$$.

Step 1: Replace $R_i$ by $R_i-R_{i+1}$, where $1 \le i \le \sigma _1-1, \sigma _1+1 \le i \le m-2,1 \le i \le \sigma _2-1$ and $\sigma _2+1 \le i \le n-2$. Then it simplifies to the new determinant, that is $\det (E)$.
Step 2: In $\det (E)$, replace $C_{i+1}$ by $C_i+ C_{i+1}$, where $2 \le i \le \sigma _1-1, \sigma _1+2 \le i \le m-1, 2 \le i \le \sigma _2-1$ and $\sigma _2+2 \le i \le n-1$. Let the new determinant be $\det (F)$.
Step 3: Expanding $\det (F)$ successively along the rows *i*, $1\le i\le \sigma _1-1, \sigma _1+1\le i\le m-2, 1\le i\le \sigma _2-1,\sigma _2+1\le i\le n-2$ . Then $\det (E)=\left( \lambda (S_{m,n})_S-\frac{2}{3}\right) ^{2\sigma _1-2}\left( \lambda (S_{m,n})_S)\right) ^{m+n-2\sigma _1-4}\det (H)$, where $\det (H)=$
  $$\begin{vmatrix} \lambda (S_{m,n})_S-\frac{2}{3}&0&-\sqrt{\frac{m+1}{3m}}&0&0&0\\ 0&\lambda (S_{m,n})_S&-\sqrt{\frac{m-1}{m}}&0&0&0\\ -\sigma _1\sqrt{\frac{m+1}{3m}}&-(m-\sigma _1-1)\sqrt{\frac{m-1}{m}}&\lambda (S_{m,n})_S&0&0&-\sqrt{\frac{m+n-2}{mn}} \\ 0&0&0&\lambda (S_{m,n})_S-\frac{2}{3}&0&-\sqrt{\frac{n+1}{3n}}\\ 0&0&0&0&\lambda (S_{m,n})_S&-\sqrt{\frac{n-1}{n}}\\ 0&0&-\sqrt{\frac{m+n-2}{mn}}&-\sigma _1\sqrt{\frac{n+1}{3n}}&-(n-\sigma _1-1)\sqrt{\frac{n-1}{n}}&\lambda (S_{m,n})_S\\ \end{vmatrix}$$
  Further simplifying *ABC* characteristic polynomial of $(S_{m,n})_S$ is given by,

$$\left( \lambda (S_{m,n})_S-\frac{2}{3}\right) ^{2\sigma _1-2}\left( \lambda (S_{m,n})_S)\right) ^{m+n-2\sigma _1-4}Q(\lambda (S_{m,n})_S)$$, where

$$\begin{aligned}& Q(\lambda (S_{m,n})_S)=\frac{1}{9mn}[ 9mn\lambda ^6(S_{m,n})_S-12mn\lambda ^5(S_{m,n})_S\\ & +\left( 12\sigma _1mn-12\sigma _1m-12\sigma _1n-9m^2n-9mn^2+40mn-18m-18n+18\right)\\ & \lambda ^4(S_{m,n})_S+\left( -20\sigma _1mn+14\sigma _1m+14\sigma _1n12m^2n+12mn^2-48mn+24m+24n-24\right)\\ & \lambda ^3(S_{m,n})_S+( 4\sigma _1^2mn+16\sigma _1^2-6\sigma _1m^2n+12\sigma _1m^2-6\sigma _1mn^2+32\sigma _1mn-34\sigma _1m\\ &+12\sigma _1n^2-34\sigma _1n+24\sigma _1+9m^2n^2-22m^2n+9m^2-22mn^2+52mn-26m+9n^2-26n+17)\\ & \lambda ^2(S_{m,n})_S+( -8\sigma _1^2mn-8\sigma _1^2m-8\sigma _1^2n-16\sigma _1^2+10\sigma _1m^2n-14\sigma _1m^2+10\sigma _1mn^2-40\sigma _1mn+38\sigma _1m-14\sigma _1n^2\\ &+38\sigma _1n-28\sigma _1-12m^2n^2+24m^2n-12m^2+24mn^2-48mn+24m+24n-12)\\ & \lambda (S_{m,n})_S+( 4\sigma _1^2mn-4\sigma _1^2m-4\sigma _1^2n+4\sigma _1^2-4\sigma _1m^2n+4\sigma _1m^2-4\sigma _1mn^2\\ &+16\sigma _1mn-12\sigma _1m+4\sigma _1n^2-12\sigma _1n+8\sigma _1+4m^2n^2-8m^2n+4m^2-8mn^2+16mn+4n^2-8n+4) ] . \end{aligned}$$

$$\square$$

## QSPR analysis of some chemical compounds useful in drug preparation

The concept of topological indices has gained significant attention in chemistry as a means to establish correlations between the structural properties of chemical compounds and their experimentally determined physico-chemical characteristics. While early research mainly concentrated on hydrocarbon molecules(molecules containing only carbon and hydrogen), recent development in graph theory have expanded the scope to include hetero-atomic molecules. These molecules, which contain atoms other than carbon and hydrogen, present unique challenges and opportunities for analysis. To address this, researchers have introduced the use of graphs with self-loops to represent hetero-atoms in molecular structures. In this representation, each hetero-atom ‘x’ is replaced by a self-loop. This approach allows for a more comprehensive study of hetero-molecules, as each self-loop effectively replaces a hetero-atom in the graphical representation. By incorporating these self-loops, scientists can extend the application of topological indices to a broader range of chemical compounds, potentially uncovering new insights into structure-property relationships. The QSPR analysis plays a crucial role in modern drug discovery and development by establishing mathematical relationships between chemical structures and their physical, chemical properties and it is a powerful computational tool that accelerates drug discovery by predicting key molecular properties and improving the efficiency of drug design.

### Definition 4

D. V. Anchan et al. in^31^ proposed Sombor index $SO(G_S)$ of a graph $G_S$ and is defined as,

5 $$\begin{aligned} SO(G_S)=\sum \limits _{v_iv_j\in E(G)}\sqrt{d_i^2(G_S)+d_j^2(G_S)}+\sqrt{2}\sum \limits _{v_i\in S}d_i(G_S). \end{aligned}$$

### Definition 5

The first Zagreb index is proposed by S. S. Shetty et al. in^32^, and is defined as,

6 $$\begin{aligned} M_1(G_S)=\sum \limits _{v_iv_j \in E(G)}(d_i(G_S)+d_j(G_S))+\sum \limits _{v_i\in S}2d_i(G_S). \end{aligned}$$

### Definition 6

The Randic index $R(G_S)$ of a graph $G_S$ is proposed by A. Harshitha et al. in^33^ and defined as,

7 $$\begin{aligned} R(G_S)=\sum \limits _{v_iv_j\in E(G_S)}\frac{1}{\sqrt{d_i(G_S)d_j(G_S)}}+\sum \limits _{v_i\in S}\frac{1}{d_i(G_S)}. \end{aligned}$$

**Fig. 1:**
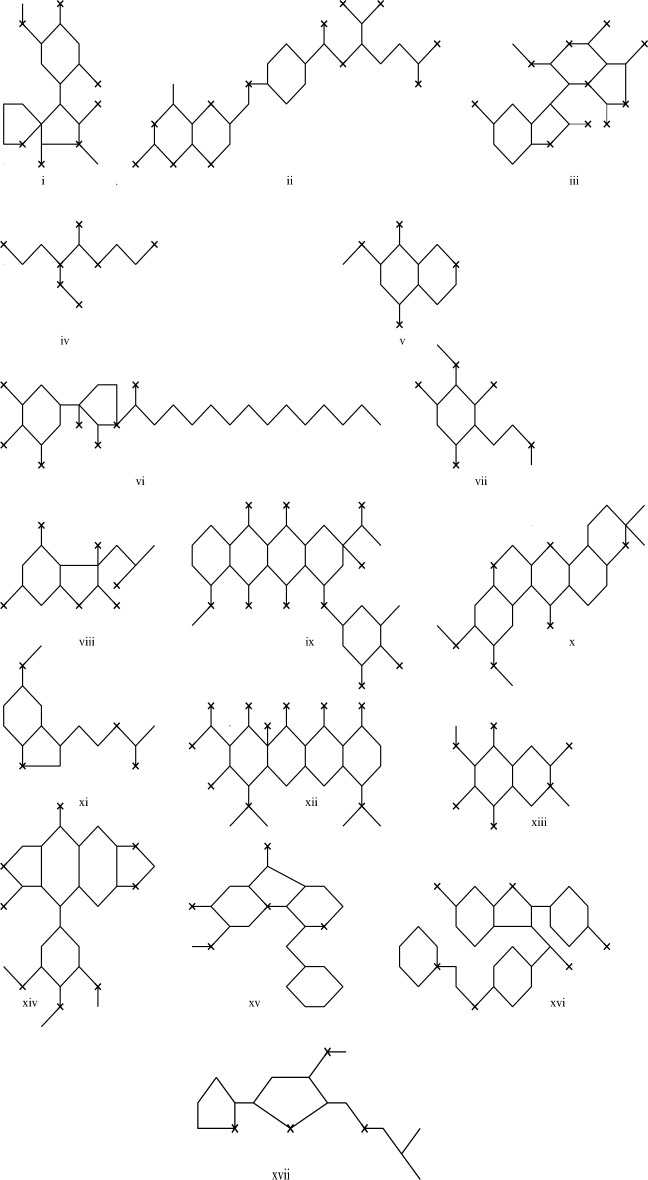
Molecular graphs with self-loops.

Here we listed degree based topological indices of a graph with self-loops for modeling *ABC* index of a graph with self-loops of anticancer drugs. The chemical compounds of anticancer drugs and the values of boiling point(BP) and molar refraction(MR) are listed in this section are taken from^34^. The topological indices listed in Table 2 for the chemical compound shown in Figure 1 are determined using edge partitioning. By counting the edges whose end vertices share the same degree type $(d_u,d_v)$, we calculate the corresponding topological indices.

**Table 1:** Chemical compounds of anticancer drugs and their physico-chemical properties.

S.No.	Drugs	BP	MR
i	Amathaspiramide E	572.7	89.4
ii	Aminopterin	782.27	114
iii	Aspidostomide E	798.8	116
iv	Carmustine	309.6	46.6
v	Caulibugulone E	373	52.2
vi	Convolutamide A	629.9	130.1
vii	Convolutamine F	387.7	73.8
viii	Convolutamydine A	504.9	68.2
ix	Daunorubicin	770	130
x	Deguelin	560.1	105.1
xi	Melatonin	512.8	67.6
xii	Minocycline	803.3	116
xiii	Perfragilin A	431.5	63.6
xiv	Podophyllotoxin	597.9	104.3
xv	Pterocellin B	521.6	87.4
xvi	Raloxifene	728.2	136.6
xvii	Tambjamine K	391.7	76.6

**Table 2:** Chemical compounds of anticancer drugs with topological indices values.

Drugs	ABC($G_S$)	SO($G_S$)	$M_1(G_S)$	R($G_S$)
Amathaspiramide E	21.0215	139.8607	194	10.6197
Aminopterin	30.741	201.2596	282	15.7705
Aspidostomide E	25.1659	175.69	246	12.295
Carmustine	11.9569	84.5573	118	5.9121
Caulibugulone E	12.3122	75.1008	104	6.7441
Convalutamide A	26.5759	152.9496	214	15.3339
Convolutamine F	13.8045	85.1035	118	7.2003
Convolutamydine A	16.2323	102.9881	144	8.3322
Daunorubicin	35.4365	219.5628	306	18.4956
Deguelin	26.1228	165.076	228	13.8205
Melatonin	15.1939	92.4609	128	8.1833
Minocycline	31.5057	205.2132	284	15.8094
Perfragilin A	16.3782	108.3798	150	8.1517
Podophyllotoxin	28.5285	185.8164	258	14.4669
Pterocellin B	21.819	135.1974	188	11.7295
Raloxifene	30.112	176.475	246	16.7672
Tambjamine K	16.642	101.4898	140	9.078

Based on the data presented in the Table 1, the $ABC(G_S)$ index shows a significant correlation with the boiling point(BP) and molar refraction(MR).

Consider the following model:

8 $$\begin{aligned} P =X(\pm S_e)(TI)+Y(\pm S_e), \end{aligned}$$

Here, P, $S_e$, X, Y and TI represent the property, standard error of the coefficients, slope, intercept and index respectively. Similarly, SE, r, F and SF denote the standard error of the model, correlation coefficient, F-test value, and significance respectively.

For anticancer drug compounds, the $ABC(G_S)$ index exhibits a stronger correlation with boiling point and molar refraction. Specifically, the following regression equations for anticancer drugs are derived using Model 8.

9 $$\begin{aligned} BP=19.577(\pm 2.355)(ABC(G_S))+132.082(\pm 55.319),\nonumber \\ r^2=0.8216, SE =70.838 , F = ,69.097, SF =5.36\times 10^{-7}. \end{aligned}$$

**Fig. 2:**
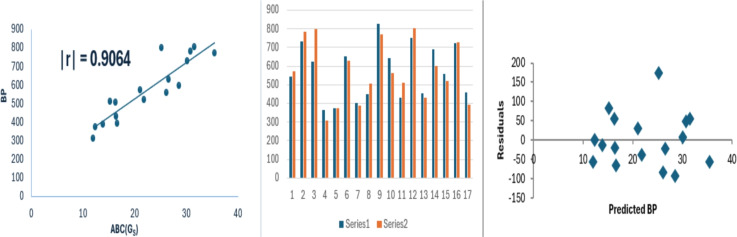
Linear relationship of $ABC(G_S)$ with BP, experimental vs predicted BP, and residual plot.

10 $$\begin{aligned} MR=3.551(\pm 0.347)(ABC(G_S))+13.508(\pm 8.151),\nonumber \\ r^2=0.8747, SE =10.44 , F =104.7 , SF =3.68\times 10^{-8}. \end{aligned}$$

**Fig. 3:**
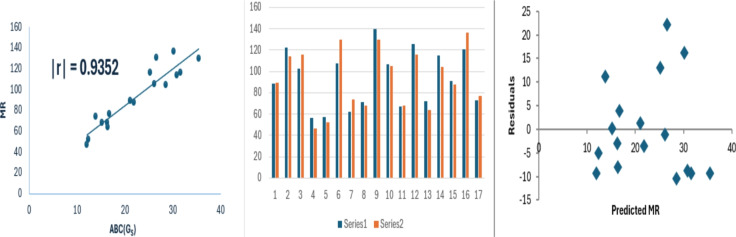
Linear relationship of $ABC(G_S)$ with MR, experimental vs predicted MR, and residual plot.

**Table 3:** Correlation coefficients of different properties with topological indices.

Index	BP	MR
ABC(G)	0.862	0.913
$ABC(G_S)$	0.9064	0.9352

In Figs. 2 and 3, the circles represent points (*x*, *y*) where *x* corresponds to $ABC(G_S)$ and *y* denotes the properties, specifically boiling point and molar refraction. The line depicted in the figures represents the regression line.

The data variance for boiling point and molar refraction is approximately 82% and 87% respectively. As the standard error value decreases, the F-value increases and the regression relationship becomes stronger. The F-value in model 10 is comparatively high. A model is considered statistically reliable when the SF value is below 0.05. In each instance, the SF value is notably lower than 0.05. Bar diagrams are used to compare the experimental properties with those predicted properties from model 8. In these figures, series 1 represents the experimental values, while series 2 shows the predicted values. The figures shows that the strong correlation between experimental results and theoretical predictions. Moreover, the random distribution of residuals around the zero line suggests that the model is consistent. To evaluate the performance of $ABC(G_S)$ in comparison with well-known degree-based indices, we analyze its correlation with the Sombor index of $G_S$, the first Zagreb index of $G_S$, and the Randić index of $G_S$.

The $ABC(G_S)$ index is noticed to perform well for Sombor index of $G_S$, frist Zagreb index of $G_S$ and Randic index of $G_S$. The corresponding regression relations are as follows:

11 $$\begin{aligned} SO(G_S)=6.2386(\pm 0.2739)(ABC(G_S))+2.3137(\pm 6.4342),\nonumber \\ r^2=0.9719, SE = 8.239, F = 518.648, SF =4.77\times 10^{-13}. \end{aligned}$$

**Fig. 4:**
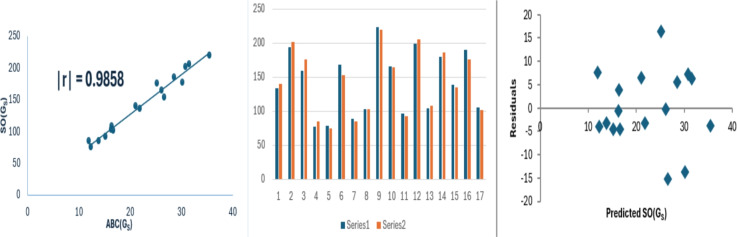
Linear relationship between $ABC(G_S)$ and $SO(G_S)$, along with experimental and predicted $SO(G_S)$ values and the residual plot.

12 $$\begin{aligned} M_1(G_S)=8.713(\pm 0.385)(ABC(G_S))+2.401(\pm 9.044),\nonumber \\ r^2=0.9715, SE = 11.58, F = 512.140, SF =5.23\times 10^{-13}. \end{aligned}$$

**Fig. 5:**
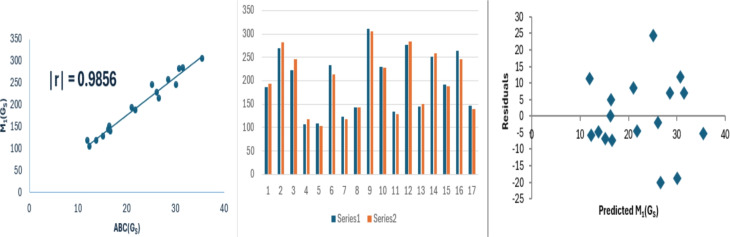
Linear relationship between $ABC(G_S)$ with $M_1(G_S)$, along with experimental and predicted $M_1(G_S)$ values and the residual plot.

13 $$\begin{aligned} R(G_S)=0.5238(\pm 0.2006)(ABC(G_S))-0.0059(\pm 0.47172),\nonumber \\ r^2=0.9785, SE = 0.6034, F = 681.822, SF =6.43\times 10^{-14}. \end{aligned}$$

**Fig. 6:**
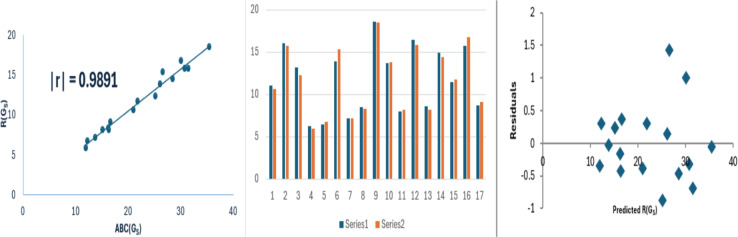
Linear relationship between $ABC(G_S)$ with $R(G_S)$, along with experimental and predicted $R(G_S)$ values and the residual plot.

The data variance for $SO(G_S)$, $M_1(G_S)$ and $R(G_S)$ is approximately 97%, 97% and 97% respectively. The model exhibits low standard errors, with model 13 showing exceptionally small values. This reduced standard error contributes to the model’s reliability and results in an increased F-value, particularly for $R(G_S)$. The SF values are significantly below 0.05. Bar diagrams are used to compare the experimental properties with those predicted properties from model 8. In these diagrams, series 1 represents the experimental values, while series 2 shows the predicted values. The Figs. 4, 5 and 6 shows that the strong correlation between experimental results and theoretical predictions. Moreover, the random distribution of residuals around the zero line suggests that the model is consistent.

In this section, the development of a QSPR model for anticancer drug chemical compounds using topological indices derived from chemical graphs. The model establishes a quantitative link between chosen topological indices and the drugs physio-chemical properties. Statistical analysis of the linear QSPR model reveals that the ABC index of a graph $G_S$ demonstrates superior correlation values for boiling point and molar refraction as shown in Table 3 with $r=0.9064$ and $r=0.9352$ respectively. The study calculates topological indices of chemical graphs for the drugs and applies them to linear QSPR models for chemical compounds. Results indicate that all models are not only significant but also optimally fitted.

In the comparison of topological indices, the *ABC* index of a graph $G_S$ was compared with the Sombor index, the first Zagreb index, and the Randic index of graphs with self-loops. Among these, the correlation between $ABC(G_S)$ and $R(G_S)$ shows the maximum correlation value of $r = 0.9891$.

## Techniques used for computation of results

We used various methods and strategies in our analysis. Specifically, we employed edge partition methodology, analytical techniques, theoretical graph utilities, and degree counting methods for our calculations. We determined the correlation coefficients between the *ABC* indices of a graph $G_S$ and several physico-chemical properties, such as boiling point (BP) and molar refraction (MR). Additionally, we calculated the correlation coefficients between the *ABC* index of graph $G_S$ and other indices, including the Sombor index $(SO(G_S))$, the first Zagreb index $(M_1(G_S))$, and the Randic index $(R(G_S))$. For these calculations, we used Microsoft Excel to streamline the analysis. We also employed MATLAB software for mathematical equations and verification purposes, while Microsoft Excel was used to create 2D graphs that compare topological indices with compound properties.

## Methodology

In the first step, we convert the chemical structure into a molecular graph. In this graph, we will replace any heteroatoms present in the chemical compound with the symbol x. Next, we will gather the physicochemical properties of the chemical compounds and estimate the values of the topological indices. For linear regression analysis, we utilized the data analysis tools in Microsoft Excel.

## Conclusion

In this work, we have extended *ABC* index of a graph *G* to a graph $G_S$ and defined a *ABC* energy of a graph $G_S$ and we obtained upper bounds spectral radius of a graph $G_S$ and some bounds for $E_{ABC}(G_S)$ and $ABC(G_S)$ in terms of *m*, *n*, $\Delta$ and $\delta$. Also, we computed the *ABC* energy for complete graph, cocktail party graph and crown graph with self-loops. We also derived the characteristic polynomial of double star graph with self-loops. We explored the correlation between $ABC(G_S)$ and various physico-chemical properties, such as boiling point (BP) and molar refraction (MR). Additionally, we established correlations between $ABC(G_S)$ and specific indices, namely the Sombor index $(SO(G_S))$, the first Zagreb index $(M_1(G_S))$, and the Randic index $(R(G_S))$. In QSPR analysis of some chemical compounds with self-loops gave a better correlation as compared to a simple graph correlation. Form this study, the following conclusions are made:

- The *ABC* index of a graph with self-loops shows better correlation with boiling point and molar refraction than the *ABC* index of graph *G*.
- Comparing the *ABC* index of the graph $G_S$ with $SO(G_S)$ reveals a strong correlation of $r=0.9858$.
- Comparing the *ABC* index of the graph $G_S$ with $M_1(G_S)$ shows a strong correlation of $r=0.9856$.
- Comparing the *ABC* index of the graph $G_S$ with $R(G_S)$ shows a strong correlation of $r=0.9891$.
- In the comparison of topological indices, the *ABC* index of a graph $G_S$ was analyzed alongside the Sombor index, the first Zagreb index, and the Randic index of graphs with self-loops. Among these indices, the correlation between $ABC(G_S)$ and $R(G_S)$ exhibited the highest correlation value, with $r = 0.9891$.

The degree-based topological indices such as second Zagreb index, Harmonic index, atom bond sum connectivity index, sum-connectivity index and many more for simple graphs have been studied and one can study all these indices for graph with self-loops.

## Data Availability

All data generated or analysed during this study are included in this published article.
